# Beaming random lasers with soliton control

**DOI:** 10.1038/s41467-018-06170-9

**Published:** 2018-09-21

**Authors:** Sreekanth Perumbilavil, Armando Piccardi, Raouf Barboza, Oleksandr Buchnev, Martti Kauranen, Giuseppe Strangi, Gaetano Assanto

**Affiliations:** 1 Laboratory of Photonics, Tampere University of Technology, FI-33101 Tampere, Finland; 2 NooEL—Nonlinear Optics and OptoElectronics Lab, University ”Roma Tre”, IT-00146 Rome, Italy; 3 Optoelectronics Research Centre, University of Southampton, SO17 1BJ Southampton, UK; 4 Department of Physics, Case Western Reserve University, Cleveland, OH 44106-7079 USA; 5 CNR-NANOTEC & University of Calabria, 87036 Rende, Italy; 6 CNR-ISC, IT-00185 Rome, Italy

## Abstract

Random lasers are resonator-less light sources where feedback stems from recurrent scattering at the expense of spatial profile and directionality. Suitably-doped nematic liquid crystals can random lase when optically pumped near resonance(s); moreover, through molecular reorientation within the transparency region, they support self-guided optical spatial solitons, i.e., light-induced waveguides. Here, we synergistically combine solitons and collinear pumping in weakly scattering dye-doped nematic liquid crystals, whereby random lasing and self-confinement concur to beaming the emission, with several improved features: all-optical switching driven by a low-power input, laser directionality and smooth output profile with high-conversion efficiency, externally controlled angular steering. Such effects make soliton-assisted random lasers an outstanding route towards application-oriented random lasers.

## Abstract (web summary)

Owing to their lack of a conventional cavity, random lasers typically do not emit a defined beam in a specific direction. Here, the authors combine spatial solitons and collinear pumping to achieve light-confined random lasing with a smooth output profile and a controllable direction of emission.

## Introduction

Random lasers (RL) are cavityless light sources^1^, versatile in geometry, wavelength, and potential applications ranging from imaging to cancer diagnostics^2–4^, but such advantages are accompanied by poor spatial characteristics of the emitted light^5^. The quest for output directionality and improved profile^6^ was previously addressed using, e.g., fibers^7,8^, microchannels^9^, tailored pump^10^, and nanostructures^11^.

Weakly scattering nematic liquid crystals (NLC) with organic dopants were used earlier for RL in thin cells, capillaries and free-standing films, exploiting thermal and electro-optic responses to adjust the emission wavelength and the efficiency^12–21^. NLC consist of elongated molecules with orientational order along the molecular director (optic-axis) **n**. Its thermal dynamics, through fluctuations in the local dielectric tensor, cause anisotropic light scattering^22^, which can yield weak light-localization and feedback for lasing in the presence of optical amplification. Common NLC also exhibit birefringence and a large reorientational optical nonlinearity^23^, which supports self-guided beams—nematicons—of linearly polarized light at mW-powers and over mm-propagation distances^24^. Owing to the nonlocal response of such soft-matter, nematicons are stable three-dimensional optical spatial solitons^25^; in uniaxial NLC they are extraordinary waves—which walk-off with the Poynting vector at a few degrees with the wave-vector (Methods)^26^—and operate as light-induced graded-index channels for co-polarized signals of arbitrary wavelengths^24^.

Hereby, we demonstrate that in dye-doped liquid crystals a suitable combination of passive nonlinear optics—namely near-infrared nematicons through self-focusing- and light-matter interaction—namely lasing in random media with optical pumping—can resolve a few crucial issues of RL. The investigated soliton-enhanced random laser is highly efficient and exhibits a smooth and directional emission, with output angle controlled by external stimuli such as voltage; it can also be turned off/on by a low-power near-infrared input. These novel features can potentially boost the impact of soft-matter RL on photonics as well as other areas where cavityless/tunable lasers are potentially relevant, including cancer therapy, sensing, and speckle-free imaging.

## Results

### Nematicon-assisted random laser in liquid crystals

**Fig. 1:**
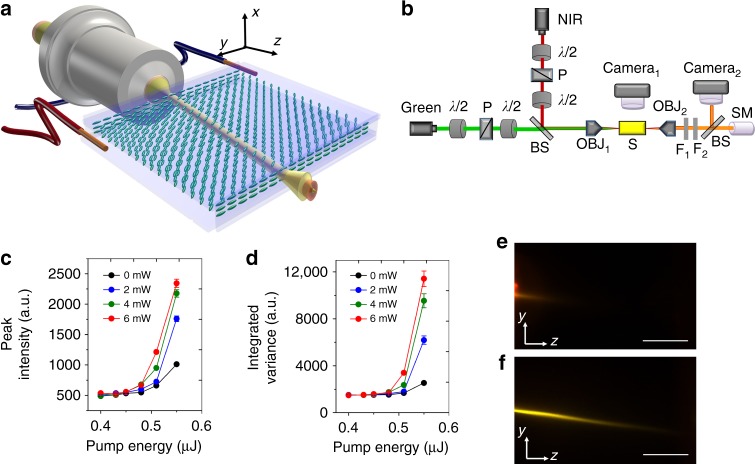
Configuration and basic features of nematicon-assisted random laser. **a** Planar cell configuration and orientation. Here, the two wires allude to the application of a bias voltage across the cell thickness (see Methods). The molecular director distribution **n** (optic-axis) is represented by blue ellipses. **b** Sketch of the basic experimental setup, with green pulsed pump and continuous-wave near-infrared (NIR) lasers, microscope objectives (OBJ) to inject and collect light, beam splitters (BS), polarisers (P), waveplates (*λ*/2), notch filters (F_1_ and F_2_), spectrometer (SM). **c** Typical input–output random laser characteristics for various soliton powers and an ordinary-wave pump, from the mean peak intensity. The legend indicates the input power of the near-infrared nematicon. **d** As in **c** but from the integrated variance $\sigma _I^2$ of the averaged spectra (see Eq. (9) in the Methods) over 200 gated acquisitions (in 100 ms windows), carrying out the analysis detailed in the Methods. The error bars in **c** and **d** correspond to standard deviation over 200 acquisitions. **e** Photograph of emitted random laser light in the *yz* plane for a pump energy *E* = 0.55 μJ, without nematicon. **f** As in **e** but in the presence of a 6 mW nematicon (the near-infrared was filtered out). The scale bars indicate a length of 500 μm

We used the NLC host E7 with 0.3 wt% pyrromethene 597 (guest dye) in planar cells to obtain random lasing in the initial (input) region of a near-infrared nematicon. Figure 1a, b shows the sample geometry and experimental setup. To generate a soliton in a 100 μm-thick 2 mm-long planar cell containing the dye-doped NLC, a continuous-wave (cw) beam at 1.064 μm was launched along *z* with electric field linearly polarized in the principal plane *yz*; **n** was aligned in the same plane at 45° with respect to *z* as to maximize the optical nonlinearity (i.e., reduce the power supporting the formation of nematicons)^24,27^. To pump the guest–host medium, an ordinary wave (with electric field //*x*) 6 ns-pulse beam at wavelength 532 nm was co-injected along *z*. In this configuration with molecular orientation defined by boundary conditions at the interfaces (see Methods), a pump beam with linear polarization orthogonal to **n** yielded the highest conversion rate to fluorescence and forward RL emission^28^. Pump pulse-duration, repetition-rate (20 Hz) and energy prevented significant reorientational and/or thermal nonlinear effects. Green and NIR beams were focused in the same position with comparable waists of 3 μm, i.e., a power density of 35.4 μW/μm^2^ per 1 mW cw NIR input and a power density of 5.9 W/μm^2^ per 1 μJ green input pulse. The resulting fluorescence and stimulated emission in the visible spectrum (around 580 nm) were co-polarized with—and therefore confined by—the nematicon. Owing to birefringent walk-off, the nematicon propagated in *yz* at an angle of about 7° off *z*, see Fig. 1f. The soliton was not only able to collect and guide fluorescence and stimulated emission due to pumping within the guest–host absorption spectrum, but also to affect the evolution of lasing modes in the longitudinally extended volume^5^, enhancing both RL directionality and profile. While some preliminary results on RL slope efficiency and spectral narrowing were reported earlier^28,29^, some salient basic features of soliton-aided RL are presented in Fig. 1c-f with reference to samples investigated hereby.

Due to randomness of the system and the substantial pedestal provided by fluorescence and amplified spontaneous emission (ASE), the lasing peaks emerged from the latter background, as typical of RLs. We first extracted the maximum intensity counts from each acquired emission spectrum, Fig. 1c, with lasing threshold at energies around 0.48 μJ and slope efficiency clearly improved by nematicons. Such threshold could not be retrieved when calculating the average intensity ($\bar I$, Methods) integrated versus wavelength over several gated spectral acquisitions (Supplementary Figure 3). Hence, we resorted to the statistical analysis of the intensity variance $\sigma _I^2$ (sensitive to fluctuations), as summarized in the Methods. This approach yielded the in-out curves Fig. 1d, where a threshold can be readily appreciated and matches the one in Fig. 1c.

**Fig. 2:**
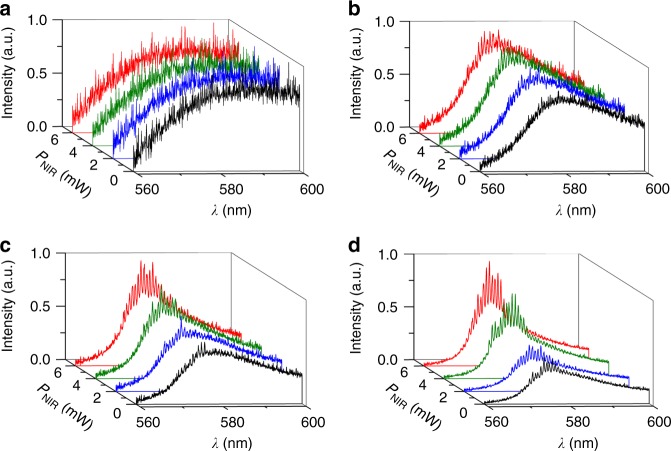
Emission spectra in the presence of nematicon. Spectral emission normalized to unity for nematicons of various powers (colors) co-launched with the ordinary-wave pump, averaged over 200 gated acquisitions. **a** Pump pulse energy *E* = 0.40 μJ. **b** *E* = 0.48 μJ. **c** *E* = 0.51 μJ. **d** *E* = 0.55 μJ

Figure 2 displays the emitted spectra averaged over several gated acquisitions and collected at the output (after propagation along *z*) with/without nematicons of various powers co-launched with the pump below/above RL threshold. This illustrates the transition from fluorescence to ASE to random lasing: the effect of the nematicon is negligible at low energies, as the waveguide simply increases the collected fluorescence or ASE^30^; its role, however, becomes pronounced at higher pump energies, contributing to narrower multihump spectra and more intense lasing peaks. The transition (vs. energy) from the ASE stochastic spikes to the RL lasing peaks is boosted by the all-optical waveguide, which affects multiple scattering and shrinks the overall spectra in the pumped region near the sample input. As observed in ref.^5^ and further reported in ref.^21^, the lasing peaks emerging from various realizations presented random amplitudes, but remained regularly spaced vs. wavelength when averaging over large numbers of pump shots (up to 200 gated measurements in 100 ms windows). Their separation suggests an equivalent cavity ≈130 μm long, comparable to the extent of the conical region where the pump is absorbed, as observed (via out-of-plane light diffusion) from above the cell (Fig. 1e). This appears consistent with weak scattering close to the regime of under-coupling (i.e., a cavity much shorter than the nematicon)^5^, although it was not experimentally ascertained as higher pump energies (needed to lengthen the cavity) induced bleaching and saturation^29^, to be further investigated. The negligible change in frequency spacing versus soliton power pinpoints the role of absorption in limiting the extent of the effective resonator^5^, enabling stimulated emission only in the initial fraction of the pumped volume.

### Spatial profile and efficiency of random laser

**Fig. 3:**
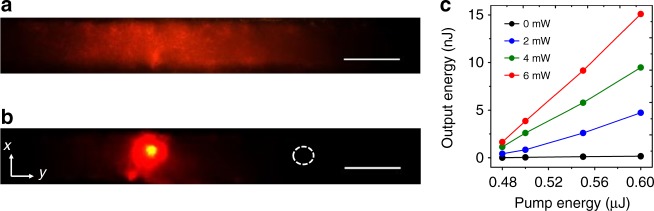
Directional random lasing. Random lasing observed at the output facet of the sample, in the plane *xy*, for pump pulses above threshold, *E* = 0.55 μJ. **a** Emitted light without a co-launched nematicon; **b** emitted light in the presence of 6 mW nematicon (the near-infrared was filtered out). The white dashed circle indicates the position of the input beam, launched with wave-vector parallel to *z*. The lateral displacement of the random laser spot is due to soliton walk-off in the plane *yz*. The scale bars refer to a length of 100 μm. **c** Output random laser energy versus pump energy/pulse above threshold for various near-infrared nematicon powers. The measured values are averaged over 200 samples and corrected for Fresnel reflection losses due to filters, lens, and cell output facet

The transversely localized RL spikes within the NIR nematicon tend to evolve along the waveguide, resulting in smoother profiles than the hot-spots commonly observed in thin samples^12,16,17^. Figure 3 shows typical RL output profiles, without/with a co-propagating nematicon. In the first case, RL light yields a diffuse pattern with low intensity; in the second case, instead, the emitted radiation remains essentially confined within the NIR-induced light channel. In essence, the (nonorthogonal) RL modes evolve and walk-off along the extraordinary wave soliton (Fig. 1f), interacting within the all-optical waveguide and roughly resulting in a bell-shape^31^. Conversely, in backward-emission experiments with light collected as it back-propagated out of the bulk toward the input, typical RL profiles were qualitatively different and exhibited several hot-spots (Supplementary Figure 5). Exploiting the achieved beaming of this random laser, in Fig. 3c we present the measured output energy vs. pump input energy above threshold, for various NIR nematicon powers. The measured values, averaged over 200 shots and corrected for Fresnel losses of the pump at the input interface (≈4%), Fresnel losses of the emitted light at the output interface (≈4%), and at the optical elements (≈17.6%) before the detector (F_1_, F_2_, OBJ_2_ in Fig. 1b), yield input/output conversion efficiencies as high as 2.62% when pumping at 0.6 μJ/pulse and co-launching NIR solitons at 6 mW, a substantial figure when considering the propagation losses over the 2 mm-long sample. Assuming scattering and absorption losses close to 6.95 cm^−1^ at visible wavelengths in the host NLC^32^, in the limit of a random laser with soliton propagation within the actively pumped region of ≈130 μm (see above), then the extrapolated conversion efficiency reaches a remarkable 9.62%. Such a high figure in organic soft-matter compares well with those measured in Sulforhodamine B with TiO_2_^33^.

### Transistor-like operation of nematicon random laser

**Fig. 4:**
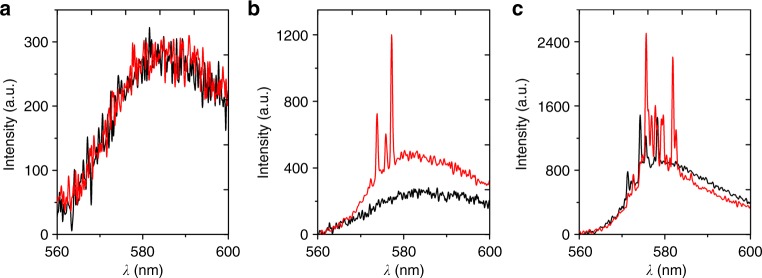
Soliton switching of random laser. Emission spectra gated over 100 ms, obtained without (black lines) and with a co-launched 6 mW nematicon (red lines) when the pump energy per pulse (in the absence of near-infrared input) is **a** below threshold (*E* = 0.45 μJ), **b** almost at (*E* = 0.48 μJ), **c** above threshold (*E* = 0.55 μJ)

Remarkably, in nematicon-assisted RL we could all-optically increase photon collection as well as pump–matter interaction and bring the system above threshold, turning-on the laser with continuous-wave NIR. Figure 4 shows 100 ms-gated emission spectra collected for pump energies well-below, almost at and above threshold in the presence of (red lines) or without (black lines) a 6 mW NIR nematicon, respectively. When injecting the latter in the system below threshold, no appreciable changes could be observed in the spontaneous emission (Fig. 4a); above threshold (Fig. 4c) the nematicon induced spectral shift and narrowing, with the appearance of more numerous and intense lasing peaks; almost at threshold (Fig. 4b) the soliton switched-on the RL, demonstrating a remarkable means to modulate the RL operation by a low-power nonresonant input and so realize a random transistor-laser (see also Supplementary Figure 4). Noteworthy, this all-optical switching does not rely on absorption and photothermal response^19^, but only on the linearly polarized NIR beam able to launch reorientational solitons.

### Angular steering of the random laser emission

**Fig. 5:**
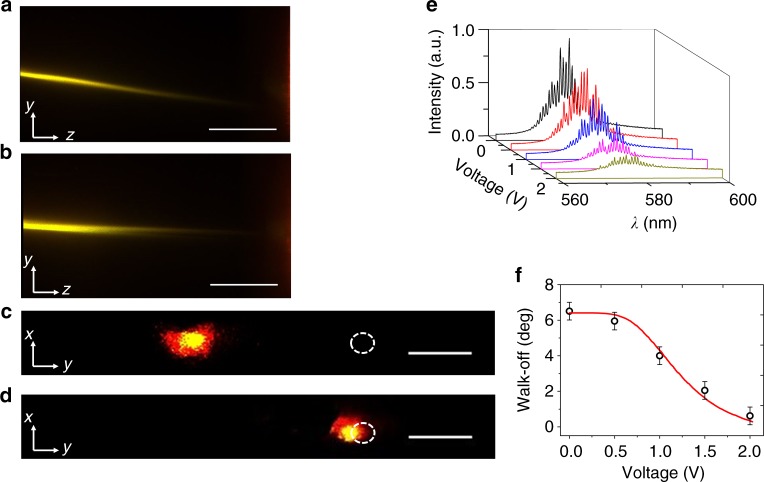
Controlled steering of soliton-assisted random laser emission. **a** Random lasing within a 5 mW near-infrared soliton, with walk-off at 7° in the plane *yz* in the absence of voltage bias. **b** Random lasing within a 5 mW near-infrared soliton, with vanishing walk-off at a bias *V* = 2 V. The scale bars in **a** and **b** indicate a length of 500 μm. **c** Output random laser spot in *xy* for *V* = 0 V, corresponding to **a**. **d** Output random laser spot in *xy* for *V* = 2 V, corresponding to **b**; the white dashed circles in **c** and **d** indicate the direction of the input wave-vector, the scale bars correspond to 100 μm. **e** Random laser emission spectra acquired at the 5 mW nematicon output for various applied voltages. **f** Measured (symbols with error bars, s.d.) and calculated (solid line) walk-off versus applied voltage (see Eq. (5) in the Methods). The error bars (in s.d. units) are computed over 25 acquisitions and the pump energy per pulse is well above threshold

Nematicons are light-induced waveguides whose trajectories can be deviated by external stimuli acting on the director distribution^24^. This allows steering the directional RL emission at will and beaming it to various output destinations. To demonstrate this in a simple case, we used thin film electrodes to apply a voltage across the cell thickness *x* in order to alter the principal plane *yz* of soliton propagation and its observable walk-off^26^. Figure 5a, b compares the RL emitted streaks along the observation plane *yz* for *V* = 0 V (Fig. 5a) and *V* = 2 V (Fig. 5b), respectively, when pumping above threshold. As *V* increases, the director realigns toward *x* and the nematicon changes direction and moves toward an ordinary-wave configuration with Poynting vector **S**//**k**//**z**, with a vanishing walk-off (Supplementary Figure 2). Although the vector **S** evolves on the surface of a cone (with axis in *yz* and apex angle defined by the initial walk-off, see Methods and Supplementary Figure 2), the RL streak gets steered from 7° to nearly 0°, as graphed in Fig. 5f, with an overall *y*-displacement of about 245 μm at the output. The photographs in Fig. 5c, d show the RL output profile for *V* = 0 (Fig. 5c) and *V* = 2 V (Fig. 5d), respectively. The RL light diffusing out of the soliton at high voltage is attributed to the interplay of electro-optic reorientation and scattering at the cell entrance where boundaries play a stronger role; moreover, as the fixed-power nematicon evolves versus voltage towards an ordinary-wave configuration (**S**//**k**), it becomes progressively less confined/confining as the associated waveguide has a lower index contrast. Correspondingly, the emitted RL spectra exhibit lower peak intensities, as apparent in Fig. 5e. Despite the fact that the angular steering was not optimized for in-plane re-addressing, these results demonstrate a directional RL emission, with pointing control adjusted by external stimuli such as voltage. Effective RL beaming can also be achieved by adopting alternative strategies to modify the nematicon trajectory within the principal plane *yz* of propagation. These include inter-digitated electrodes on the planar interfaces^34^, external light beams altering the director distribution^24^, magnetic fields^35^. The latter approach, in particular, allows doubling the overall angular swing by rotating the magnetic field in *yz* around *x*.

## Discussion

We have designed, investigated, and reported a random laser in light-sculpted dye-doped NLC, whereby the synergy of the nonresonant reorientational nonlinear response with optical gain and weak scattering enables cavityless soliton-enhanced laser emission. Besides the high-conversion efficiency and spectral narrowing, this soft-matter laser system entails all-optical low-power switching, emission directionality with smooth profile and pointing control by external stimuli, thereby introducing soliton-adjustable NIR-controlled RL.

These results demonstrate the benefits of suitably combining diverse nonlinear optical responses; they also solve some RL vexing issues, specifically poor directionality and profile. We foresee this synergistic route to impact on practical resonator-less lasers in disordered weakly scattering soft-media.

## Methods

### Samples

Planar glass cells, 100 μm thick (*x*) 30 mm wide (*y*) and 2 mm long (*z*), were prepared with Borosilicate glass slides equipped with Indium Tin Oxide (ITO) thin film electrodes; the slides were subsequently spin-coated with polyimide and mechanically rubbed to ensure planar anchoring of the molecular director in the plane *yz* at 45° with respect to *z*, with a slight pretilt ≤2° to prevent the insurgence of the Freedericksz transition threshold and its angular degeneracy^36^ vs. applied voltage. Spacers were inserted to define the thickness. Glass microscope cover slides 160 µm thick were glued orthogonally to *z* in order to seal the cell and so define input and output facets. These extra interfaces prevented the formation of NLC menisci and uncontrolled light depolarization; they were also coated with polyimide and rubbed along *y* to optimize the coupling of *y*-polarized electric fields to extraordinary waves in the NLC^37^. A mixture of commercial E7 (Merck) and 0.3 wt% Pyrromethene 597 dye (Exciton) was introduced to fill the cell by capillary action. For the host medium at 1.064 μm the elastic constants for splay and twist deformations are *K*_1_ = 11.7 pN and *K*_3_ = 19.5 pN, respectively; the refractive indices along the principal axes are $n_{\Vert}=1.71 \, {\rm and}\, n_{\bot}$ = 1.52.

### Experimental setup

A frequency-doubled Nd:YAG laser operating in Q-switch at 20 Hz, 532 nm, and 6 ns pulses was the pump source; a NIR continuous-wave laser at 1.064 μm was the spatial optical soliton source. Waveplates, polarisers and a microscope objective were employed to focus and co-launch the orthogonally polarized (green and NIR) beams in the midplane (*x* = 50 μm) of the cell, avoiding reflections from upper and lower interfaces. Fluorescence and laser emission, as well as the NIR beam were imaged by NLC light scattering out of the observation plane *yz* and also in the transverse plane *xy* at the cell output, using optical microscopes and high-resolution CMOS cameras. A fiber-equipped spectrometer (Ocean Optics) was used to collect and spectrally resolve the emitted light, with a resolution of 0.15 nm; a Si-detector allowed measuring the random laser emission. Additional filtering elements were introduced whenever appropriate to eliminate pump and NIR light (see also Fig. 1b). A voltage bias from a 1 kHz generator could be applied across the cell thickness through the ITO electrodes. The AC voltage supply prevented undesired NLC convection and static charge effects.

### Model

In the reference system *xyz* indicated in Fig. 1, the propagation of extraordinary polarized light beams in an anisotropic nonlinear material obeys a nonlinear Schrödinger-like equation:

1 $$2in_{\mathrm{e}}\left( {\theta _0} \right)k_0\left[ {\frac{{\partial A}}{{\partial z}} + \tan \delta \left( {\theta _0} \right)\frac{{\partial A}}{{\partial y}}} \right] + D_{{y}}\frac{{\partial ^2A}}{{\partial y^2}} + k_0^2\Delta n_{\mathrm{e}}^2\left( {\theta _0,\phi } \right)A = 0$$

where *A* is the magnetic field envelope in the slowly varying amplitude approximation, *k*_0_ the vacuum wave number, $\delta \left( {\theta _0} \right) = \arctan \left[ {\frac{{\varepsilon _{\mathrm{a}}\sin \left( {2\theta _0} \right)}}{{\varepsilon _{\mathrm{a}} + 2\varepsilon _ \bot + \varepsilon _a\cos \left( {2\theta _0} \right)}}} \right]$ the walk-off angle ($\varepsilon _{\mathrm{a}} = n_{||}^2 - n_ \bot ^2$ is the optical anisotropy) at the orientation *θ*_0_ between **k** and **n** at rest, *D*_*y*_ the diffraction coefficient along the *y* direction (sample width), and $\Delta n_{\mathrm{e}}^{}\left( {\theta _0,\phi } \right)$ the extraordinary wave refractive index potential well due to the nonlinear reorientation *ϕ*. The latter reorientation can be evaluated from the Euler–Lagrange equation^23,24,38^ modeling the competing contributions of the driving field (light beam in our case) and the restoring elastic forces in the NLC, resulting in the reorientation equation:

2 $$\nabla ^2\theta + \frac{{\varepsilon _0\varepsilon _{\mathrm{a}}Z_0^2}}{{4K\;n_{\mathrm{e}}^2(\theta _0)\cos ^2\delta }}\sin \left[ {2\left( {\theta - \delta } \right)} \right]\left| A \right|^2 = 0$$

where *θ* = *θ*_0_ + *ϕ*, *K* is the elastic Frank constant in the single elastic constant approximation (*K* ≈ *K*_1_ ≈ *K*_3_ ≈ 12 pN), *ε*_0_ the vacuum dielectric constant, *Z*_0_ the vacuum impedance. Thus, starting from *θ*_0_ and the forcing field, Eq. (2) determines the director orientation and the refractive index distribution to be considered in Eq. (1).

Equations (1) and (2) do not have—in general—exact soliton solutions^27,39^, but when integrated numerically with proper boundary conditions, they support reorientational spatial solitons in NLC—nematicons—with a nondiffracting transverse profile corresponding to the normal mode of the nonlocal graded-index waveguide described by $\Delta n_e^{}\left( {\theta _0,\phi } \right)$^24,40^. An example of the calculated evolution and profile of a near-infrared nematicon with the corresponding graded-index waveguide is provided in Supplementary Figure 1.

The Euler–Lagrange equation for the molecular director^38^, combined with the divergence-free condition for the electric displacement field, allows one to evaluate the voltage-driven elevation angle η of the optic axis **n** from the plane *yz* of the cell:

3 $$\left[ {\left( {\varepsilon _ \bot ^{{\mathrm{LF}}} + \Delta \varepsilon _{{\mathrm{LF}}}} \right)\sin ^2\left( \eta \right) + \varepsilon _ \bot ^{{\mathrm{LF}}}\cos ^2\left( \eta \right)} \right]\frac{{d^2V}}{{dx^2}} + \Delta \varepsilon _{{\mathrm{LF}}}\sin \left( {2\eta } \right)\frac{{d\eta }}{{dx}}\frac{{dV}}{{dx}} = 0$$

4 $$\frac{{d^2\eta }}{{dx^2}} + \frac{{\varepsilon _0\Delta \varepsilon _{{\mathrm{LF}}}}}{{2K}}\left( {\frac{{dV}}{{dx}}} \right)^2\sin (2\eta ) = 0$$

with *V* the applied low-frequency (LF) bias, $\varepsilon _ \bot ^{{\mathrm{LF}}}$and $\Delta \varepsilon _{{\mathrm{LF}}}$ the dielectric constant (for electric field perpendicular to the optic-axis) and the dielectric anisotropy, respectively. In this case the integration is performed along the direction *x* of the applied electric field and the resulting angular distribution of the molecular director **n** provides the refractive index landscape in which the beam propagates^24,26^.

As the voltage is uniformly applied between the planar parallel (ITO) electrodes, the molecular director **n** is pulled out of the alignment plane *yz* toward the vertical axis *x*, resulting in a rotation of the principal plane. Note that the injected nematicon remains an extraordinary wave as it adiabatically evolves in polarization through the transition region close to the input interface^41^.

For a given *η*(*V*), the corresponding angle between **k** and **n**(*V*) becomes $\psi = \arccos (\cos \eta \cos \theta _0)$, with the consequent angular steering of the Poynting vector **S** of the NIR soliton (yellow arrow in Supplementary Figure 2) and of the guided random laser emission, as seen in Fig. 5. It is straightforward to derive the projection of the voltage-dependent Poynting vector on the observation plane *yz* and compute the solid curve of the observed walk-off:

5 $$\alpha = \arctan \left( {\frac{{\tan \delta \cos \eta }}{{\sqrt {1 + \sin ^2\eta \cot ^2\theta _0} }}} \right)$$

as plotted in Fig. 5f.

### Statistical analysis of the emission spectra

Random lasing emission in an extended system exhibits characteristic fluctuations. Specifically, the lasing modes emerge from the fluorescence/ASE background as spectral peaks at certain wavelengths (see, e.g., Fig. 2, Fig. 4, Supplementary Figure 4), where the intensity varies from shot to shot. The onset of lasing is therefore associated to deviations of the intensity from its average at a given wavelength and can be quantified by the corresponding variance^42^.

To analyze the emission properties of the NLC random laser, we calculate the average intensity and the variance over *N* output spectra, defined as

6 $$\bar I\left( \lambda \right) = \frac{1}{N}\mathop {\sum}\limits_{k = 1}^N {I_k} \left( \lambda \right),$$

7 $$\sigma _I^2\left( \lambda \right) = \frac{1}{{N - 1}}\mathop {\sum}\limits_{k = 1}^N {\left( {I_k\left( \lambda \right) - \bar I\left( \lambda \right)} \right)^2} ,$$

respectively, at a specific wavelength *λ*. It is important to note that $\sigma _I^2\left( \lambda \right) = 0$ only for a constant spectrum over its recorded values. Sample data of the quantities (6) and (7) above are shown in Supplementary Figure 4 vs. wavelength for various pump energies and nematicon powers.

Furthermore, in order to gain insight on the global operation of the random laser, the average intensity and the variance can be integrated over the spectrometer response window. This approach allows one to study the system behavior over the whole spectral window $\Delta \lambda = \lambda _{\max } - \lambda _{\min }$, measuring the integrated average intensity and integrated variance, respectively, defined as:

8 $${\bar {I}} = \frac{1}{{\Delta \lambda }}\int_{\lambda _{\min }}^{\lambda _{\max }} {\bar I\left( \lambda \right)} {\mathrm d}\lambda ,$$

9 $$\sigma _I^2 = \frac{1}{{\Delta \lambda }}\int_{\lambda _{\min }}^{\lambda _{\max }} {\sigma _I^2} \left( \lambda \right){\mathrm d}\lambda .$$

Since well-defined spikes above the fluorescence/ASE pedestal characterize the transition to lasing threshold, another useful parameter is the mean intensity maximum computed as:

10 $$\bar I_{\max } = \frac{1}{N}\mathop {\sum}\limits_{k = 1}^N {\max _\lambda } I_k\left( \lambda \right).$$

As random lasing cannot be completely separated from ASE and fluorescence contributions, the integrated average intensity $\bar I$ (plotted in Supplementary Figure 3) does not exhibit a clear threshold even after the laser onset^42^, despite the presence of spikes above a certain pump level (see Fig. 2). The integrated variance Eq. (9), however, is remarkably sensitive to the emergence of spectral features, including fluctuations in the lasing peaks. Hence, it manifests a threshold as it appears in the mean peak intensity $\bar I_{\max }$ (see Fig. 1c, d).

## Electronic supplementary material

Supplementary Information

## Data Availability

The datasets generated and analyzed during the current study are presented in this published article (and its Supplementary Information) in aggregated form as figures/graphs and are available from the corresponding author upon reasonable request.
