# Supplementary material for: Beaming random lasers with soliton control
Source: Nat Commun. 2018 Sep 21;9:3863. doi: 10.1038/s41467-018-06170-9 (PMC6155064; doi:10.1038/s41467-018-06170-9)
Supplement: Supplementary file 1 — Supplementary Information [file 41467_2018_6170_MOESM1_ESM.pdf]

Supplementary Information

Beaming Random Lasers with Soliton Control

Sreekanth Perumbilavil, Armando Piccardi, Raouf Barboza, Oleksandr Buchnev, Martti Kauranen, Giuseppe Strangi and Gaetano Assanto

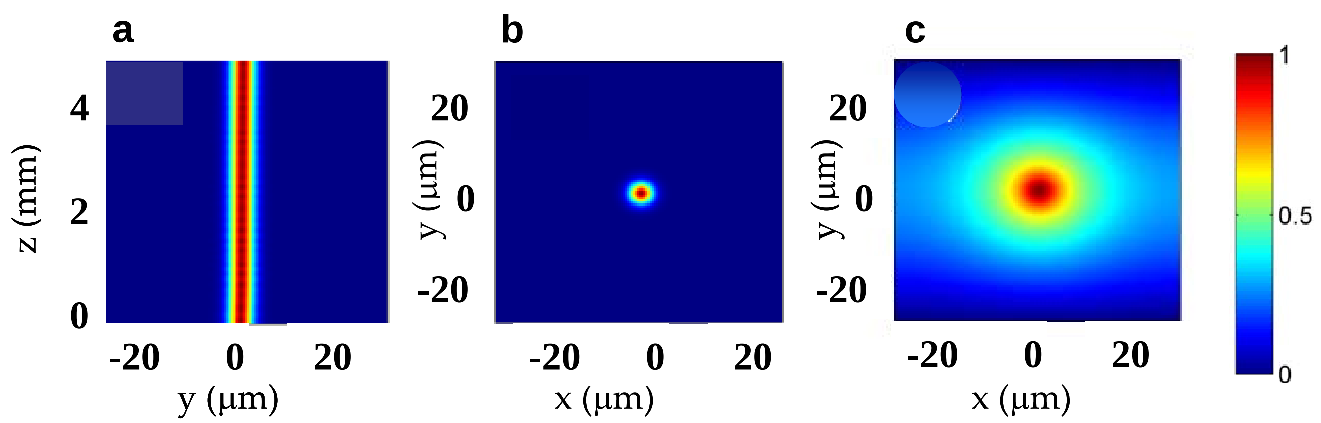

Supplementary Figure 1. Simulations of a near-infrared nematicon in standard nematic crystals

a 4 mW soliton intensity evolution in the observation plane *yz*, for typical launch conditions of a Gaussian beam of 3.6 μm waist with input wave-vector along *z* in a lossless nematic liquid crystal (E7) with molecular director **n** = n(0, π/3, π/6) at rest and thickness 60 μm. **b** Self-confined beam profile in the output transverse plane after propagation for 5 mm; **c** corresponding graded refractive index distribution in *xy*. For simplicity, linear birefringent walk-off was factored out from the model equations, hence it is not visible in these plots. The colour bar is in normalized (intensity or index) units.

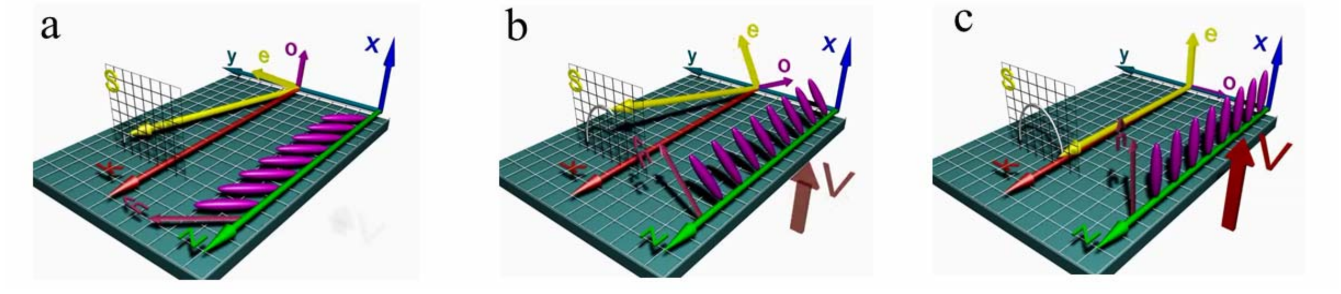

Supplementary Figure 2. Voltage-controlled steering

Artist's sketch of the angular steering produced on a nematicon beam/waveguide by the application of a voltage *V* along *x*, as the latter increases from **a** *V*=0 V to **b** *V* = 1.2 V and to **c** *V* > 2.5 V in a standard nematic liquid crystal cell with director **n** (violet arrow and ellipses) initially at 45° in the *yz* plane. The labels o and e stand for extraordinary and ordinary eigen-polarisations in he uniaxial medium. The walk-off, i. e. the angle between the Poynting vector **S** (yellow arrow) and the wave-vector **k** (red arrow), progressively reduces as *V* increases until **S** and **k** become collinear in **c**. The measurable angular steering stems from the projection of **S** in the observation plane *yz*.

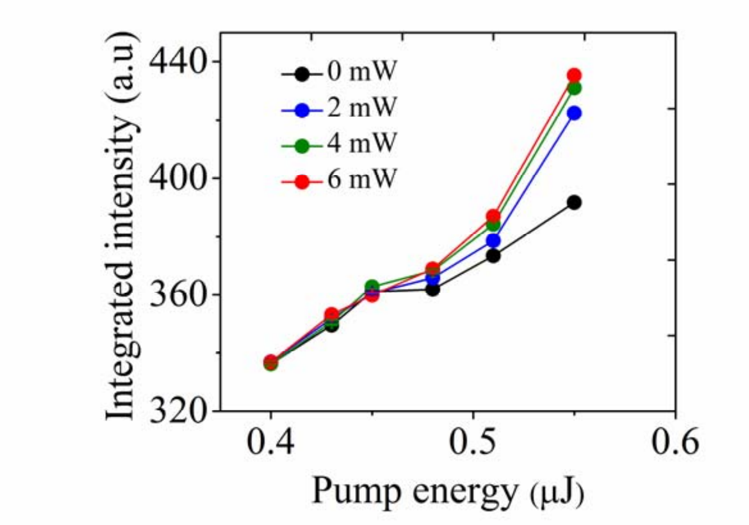

Supplementary Figure 3. Integrated average intensity $\bar{I}$ **versus pump energy for various nematicon powers** The intensity spectra of the emitted light are averaged over 200 single-shot realizations (pump pulses) and integrated versus wavelength, according to Eq. (8) in the Methods. The legend indicates the power of the launched near-infrared nematicon. No threshold can be appreciated versus pump energy (at variance with Fig. 1**c-d** in the main text).

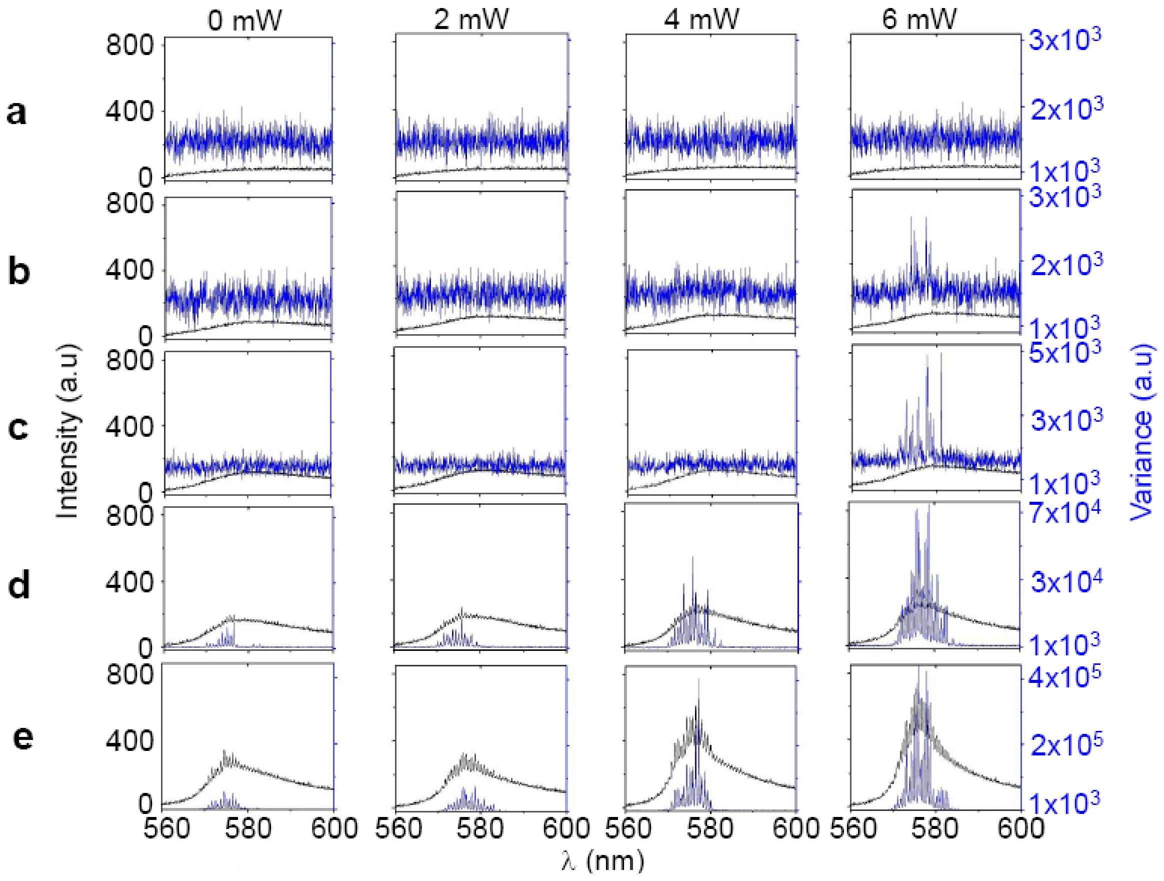

Supplementary Figure 4. Average intensity and variance of the emission spectra over 200 realizations Emission average intensity and variance are plotted versus wavelength and arranged in rows of equal pump energy and columns of equal near-infrared nematicon power. **a** Pump energy E = 0.43 μJ; **b** E = 0.45 μJ; **c** E = 0.48 μJ; **d** E = 0.51 μJ; **e** E = 0.61 μJ.

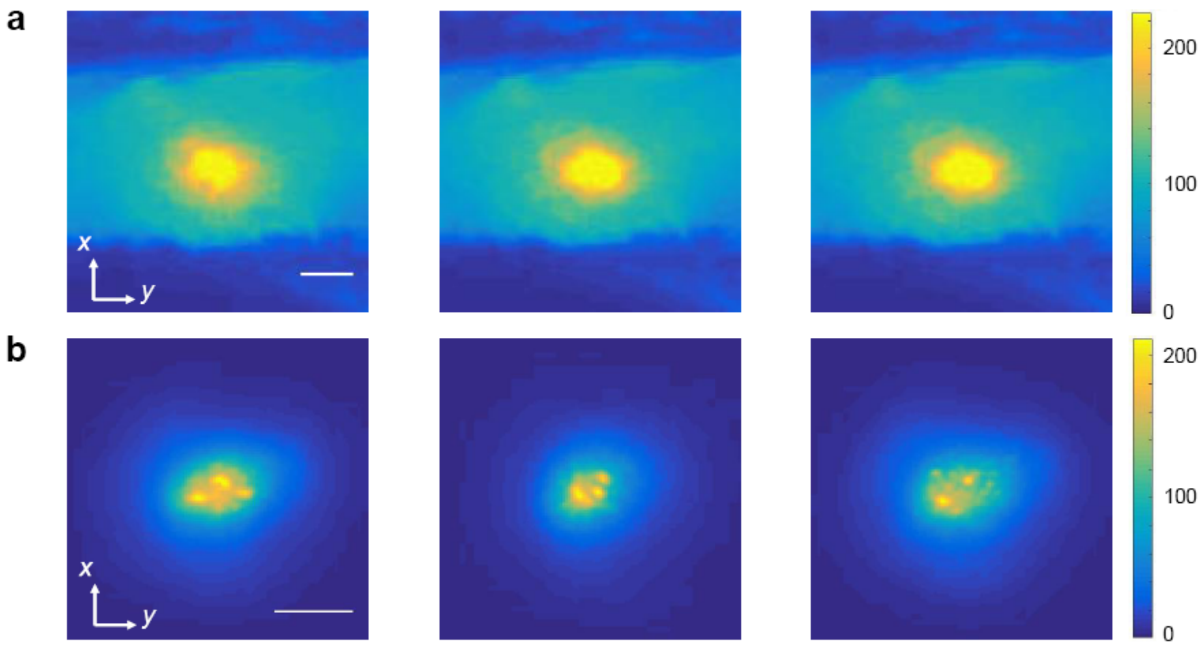

Supplementary Figure 5. Forward and backward random laser emission profiles

a Single shot realizations of forward random laser emission profile after propagation in the nematic liquid crystal cell in the presence of a near-infrared soliton. The illuminated slab corresponds to the nematic liquid crystal sample, 100 μm thick. **b** Single-shot realizations of backward random laser emission profile. The pump energy was E = 0.8 μJ and the input nematicon power P = 6 mW. The length bars in **a** and b correspond to 25 μm. The colour bars are in arbitrary intensity units.
